# Scaling law links plant growth variation to grain yield in wheat stands

**DOI:** 10.1111/nph.71028

**Published:** 2026-02-17

**Authors:** Guy Golan, François Vasseur, Yongyu Huang, Kenan Tan, Victor O. Sadras, Cyrille Violle, Thorsten Schnurbusch

**Affiliations:** ^1^ Leibniz Institute of Plant Genetics and Crop Plant Research (IPK), OT Gatersleben 06466 Seeland Germany; ^2^ The Robert H. Smith Faculty of Agriculture, Food & Environment The Hebrew University of Jerusalem Rehovot 7610001 Israel; ^3^ CEFE Univ Montpellier, CNRS, EPHE, IRD Montpellier 34090 France; ^4^ South Australian Research and Development Institute, Kaurna Country Adelaide SA 5001 Australia; ^5^ School of Agriculture, Food and Wine The University of Adelaide Adelaide SA 5064 Australia; ^6^ College of Science and Engineering Flinders University Adelaide SA 5063 Australia; ^7^ Faculty of Natural Sciences III, Institute of Agricultural and Nutritional Sciences Martin Luther University Halle‐Wittenberg 06120 Halle Germany

**Keywords:** allometry, growth rate, G×E, metabolic scaling, wheat

## Abstract

- Growth rate, a fundamental trait associated with plant resource use, scales with plant mass, following consistent allometric power laws shaped by biophysical constraints and natural selection captured in metabolic scaling theory (MST). Although well‐established in wild plants, MST has been overlooked in crop improvement.
- We quantified the relationship between individual plant mass and growth rate in 195 European winter wheat cultivars under glasshouse conditions and examined how variation in growth allometry relates to stand‐level grain yield across eight field environments and to its underlying genetic, developmental, and physiological mechanisms.
- Variation in allometry was linked to plant size, in which increased leaf allocation and faster development elevated allometric exponents. Phenotypic and genetic analyses revealed adaptive strategies, ranging from large, slow‐developing genotypes that support reproductive initiation to small, fast‐developing genotypes that enhance floret survival and reproductive effort. A shared genetic basis associated with *Photoperiod‐1* linked growth allometry in the glasshouse to genotype‐by‐environment interactions for grain yield in the field.
- Our findings demonstrate that growth allometry is biologically robust and agronomically relevant. While phenology is associated with adaptive variation in allometry, the allometric framework integrates developmental and physiological processes that jointly shape cultivar performance across environments, providing a basis for environment‐specific crop improvement.

## Introduction

Organisms acquire physical resources, matter and energy, from their environment, metabolize them, and allocate them to various functions with implications for fitness. The rate of resource uptake and the expenditure of energy and resources are largely influenced by organism size (West *et al*., 1997; Brown *et al*., 2004). Allometry describes the disproportionate change in an organism's morphology, physiology, and life‐history traits relative to changes in its size (Huxley, 1924). Allometry can be examined across various contexts: during an organism's growth and development (ontogenetic allometry), across individuals at the same developmental stage (static allometry), or across species (evolutionary allometry) (Pélabon *et al*., 2013). Allometric relationships often follow a power law, *Y* = α*X* ^β^, where trait *Y* correlates with organism mass *X*, with α as a constant and β as the allometric exponent (Huxley, 1924), typically linearized and analyzed on a log–log scale as log *Y* = log α + β log *X*.

Since Kleiber's seminal work identified a three‐fourth allometric exponent between metabolic rate and body mass across animal species (Kleiber, 1932), the pervasive nature of quarter‐power allometric scaling was observed across biological phenomena (Savage *et al*., 2004). Such scaling is mostly explained by the metabolic scaling theory (MST), which proposes that quarter‐power allometric exponents result from an equilibrium between the scaling of hydraulic transport costs and surface area for resource exchange (West *et al*., 1997). In plants, substantial evidence supports relatively invariant MST predictions concerning metabolic and growth rate, resource allocation, and life‐history traits (Enquist *et al*., 1998; Niklas & Enquist, 2001; Enquist & Niklas, 2002; Marbà *et al*., 2007). Yet, other studies suggested that allometric exponents may vary among genotypes (Vasseur *et al*., 2012, 2018), or deviate from MST predictions (Reich *et al*., 2006). Such deviations were suggested to stem from the intrinsic nonlinearity of metabolic scaling (Kolokotrones *et al*., 2010), selection of different adaptive strategies (Vasseur *et al*., 2012, 2018), environmental and physiological effects (Glazier, 2005; Coomes *et al*., 2011), or analysis focused on very small plants (Enquist *et al*., 2007; Poorter *et al*., 2015).

While MST predictions have been tested in wild plants and variations in allometric relationships have been explored, their validity and the implications of such variations for crop yield remain largely unknown. In nature, selection favors competitive phenotypes, whereas agronomic selection favors communal phenotypes that return higher yield per unit of land when grown in dense stands (Denison *et al*., 2003; Weiner *et al*., 2017; Golan *et al*., 2023). This shift in selective pressure has driven allometric scaling divergences between wild plants and crop plants; for example, Green Revolution cereals have a higher allocation to reproduction at the expense of stems that increases yield per unit land area and reduces competitive ability for light (Golan *et al*., 2024). While this illustrates changes in reproductive allocation, it remains unclear whether breeding has changed metabolic scaling in plants. Considering the evidence that variation in metabolic scaling can evolve through natural selection or genetic drift (Glazier, 2005), it is critical to know whether plant breeding for yield potential and agronomic adaptation has shifted plant allometry. Moreover, identifying links between metabolic scaling and yield may inform crop improvement through targeted breeding of allometric traits (Westgeest *et al*., 2024).

MST predicts that plant growth rate (biomass produced per time unit) scales to the three‐fourth power of plant mass (Niklas & Enquist, 2001), illustrating that plants grow relatively slower but become more resource‐efficient as they grow. In wild plants, such as *Arabidopsis thaliana*, genetic variation in growth allometry underlies a trade‐off between reproductive yield and maintenance of growth under stress, in which deviations from the three‐fourth allometric exponent reduce seed production in favor of stress tolerance (Vasseur *et al*., 2018). Similarly, leaf area allometry in wild tomato species varies around the three‐fourth allometric exponent, mediating a trade‐off between fecundity and days to wilting following drought stress (Muir & Thomas‐Huebner, 2015). If crops have a similar link between fitness components, selecting growth allometry traits could improve yield across environments.

In addition, genetic variation in growth allometry likely reflects differences in biomass allocation among plant organs (Enquist & Niklas, 2002; Golan *et al*., 2024). Biomass allocation is a dynamic process, with annual plants beginning their life cycle by investing primarily in roots and leaves. As the plant develops, allocation shifts to supporting structures such as stems and ultimately to reproduction (Harper & Ogden, 1970). These transitions are influenced by the developmental trajectory and plant size, constraining organ growth, and thus resource use and reproductive growth (Körner, 1991; Poorter *et al*., 2015; Rivera‐Amado *et al*., 2019). In cereals, grain yield is associated with the total biomass produced per area and the proportion allocated to grain, expressed as the harvest index (Donald & Hamblin, 1976). However, while the harvest index provides insights into the ultimate outcome of biomass allocation, it is a static measure that overlooks the dynamic allocation processes occurring throughout ontogeny, particularly during reproductive development, in which resource allocation is crucial for grain yield. Importantly, the harvest index does not capture size‐dependent variation in allocation of plant resources (Qin *et al*., 2013). The dynamics of floret initiation and degeneration determine inflorescence fertility in cereal crops (Huang & Schnurbusch, 2024). However, trade‐offs between reproductive and vegetative allocation (stem, root), and between structural and labile carbohydrates in the critical period of kernel set have implications for grain yield (Siddique *et al*., 1989; Rivera‐Amado *et al*., 2019; Sadras, 2021; Slafer *et al*., 2023., Huang *et al*., 2024). Therefore, a deeper understanding of allometry among organs during key reproductive developmental stages is important to understand and potentially improve grain yield.

Here, we quantified the allometric relationship between growth rate and plant mass during ontogeny of 195 European elite winter wheat (*Triticum aestivum* L.) cultivars grown under controlled conditions, with a focus on the critical phase for grain number determination (Fischer *et al*., 2024), when the number of fertile florets per spike is determined. Phenotypic analysis and genome‐wide association (GWA) scans revealed variation in allometry across genotypes, shaped by differences in developmental rate and resource allocation patterns, both of which share a common genetic basis with allometry. Moreover, we identified genetic links between reproductive development and allometry, which scaled to grain yield in independent, agronomically realistic field trials in France and Germany. Overall, our study identifies growth allometry as a physiological bridge that genetically and developmentally connects life‐history traits, reproductive strategies, and environmental adaptation.

## Materials and Methods

### Growth conditions and phenotypic measurements

Phenotypic data for growth analysis were collected from 195 hexaploid elite winter wheat (*Triticum aestivum* L.) cultivars, a subset of the GABI wheat panel (Gogna *et al*., 2022), grown under controlled glasshouse conditions at IPK Gatersleben, Germany, as previously described (Guo *et al*., 2017, 2018a,b). The investigated panel included cultivars from Germany (*n* = 93), France (*n* = 87), Denmark (*n* = 10), and the UK (*n* = 3), and one cultivar each from the Czech Republic and Austria. Grains were sown in 96‐well trays and germinated in a glasshouse under a 16 h : 8 h, light : dark photoperiod at 20°C : 16°C (day : night) for 14 d. At the two‐ to three‐leaf stage, seedlings were transferred to 4°C for 63 d of vernalization under a 10 h : 14 h, light : dark photoperiod with a light intensity of 30 μmol m^−2^ s^−1^. Following vernalization, plants underwent a 7‐d hardening period under a 12 h : 12 h, light : dark photoperiod at 15°C to allow gradual acclimation. Finally, 40 single plants from each cultivar were transplanted into 0.5‐l pots (9 × 9 × 9 cm) and assembled as groups in a temperature‐controlled glasshouse under a 16 h : 8 h, light : dark photoperiod at 20 ± 1°C : 6 ± 1°C (day : night), with minimal interference between neighboring plants. Plants were grown in a substrate containing peatmoss with 14 : 16 : 18/Nitrogen (N) : Phosphorous (P) : Potassium (K). To avoid any mineral deficiency each pot was additionally fertilized with 1.5 g of solid fertilizer (that constitute minerals 17 : 11 : 10/N : P : K). Supplemental lighting provided *c*. 250 μmol m^−2^ s^−1^ photosynthetically active radiation. Pests were controlled as needed, and plants were watered daily.

Starting 2 wk after vernalization, plants were dissected every 2 d to determine the development stage using the Kirby & Appleyard scale (Kirby & Appleyard, 1984). The developmental stages of the cultivar and the thermal time to reach the stages were assessed using the main culm of at least three randomly selected plants. A cultivar was considered to be at the Tipping (TP), heading (HD), or anthesis (AN) when at least half of the plants reached the corresponding stage. Once the plants reached a specific stage, three plants of each cultivar were randomly selected and separated for tillers and the main culm. Biomass of each component was determined as dry weight. The main culm was used to determine organ weight, spikelet number, maximum floret primordia number (usually around the green anther (GA) stage), and the percentage of floret survival (grains per spikelet at maturity/ maximum floret primordia), with traits measured at maturity (harvest index, grains per spikelet, and spikelet number) obtained from six plant replications. Thermal time (°Cd) was used to quantify growth duration to reach each stage. It was calculated as the sum of cumulative daily thermal, calculated as *T*_average_ – *T*_base_. The base temperature (*T*_base_) was assumed to be 0°C and average temperatures (*T*_average_) were calculated as ((*T*_max_ + *T*_min_)/2), referring to the maximum air temperature (°C) recorded during the light (day) period, and the minimum air temperature recorded during the dark period.

### Growth analysis

The absolute growth rate for each cultivar at each developmental stage was calculated as the whole plant aboveground biomass accumulated divided by thermal time of the growth period. Standard major axis (SMA) regressions were conducted using the smatr package (Warton *et al*., 2012) in R. The relative growth rate (RGR) between developmental stages was calculated as:

(Eqn 1) $$\mathrm{RGR}=\left(\log_{e}\;M_{2}-\log_{e}\;M_{1}\right)/\Delta t$$

where *M*_1_ and *M*_2_ are the cultivar's aboveground plant dry weight at the beginning and end of the interval, and Δ*t* is the corresponding thermal time. The averaged RGR for each cultivar across stages was extracted from a two‐parameter exponential growth model implemented in JMP17 (SAS Institute, Cary, NC, USA). The model is defined as:

(Eqn 2) $$y\left(t\right)=a\cdot e^{b\cdot t}$$

where $y\left(t\right)$ represents plant biomass at time *t*, a represents the plant's initial biomass at the terminal spikelet stage (TS), b is the estimated RGR, t is thermal time, and e is the base of natural logarithms.

Correlations between traits (*r*) were derived from the coefficient of determination (*R* ^2^) obtained after fitting linear or quadratic regressions between the respective trait pairs.

### Grain yield analysis

Grain yield for the same cultivars examined in the glasshouse was measured in plots (5–6.75 m^2^) in eight rain‐fed field experiments conducted in Germany and France during 2009–2010, as previously described (Gogna *et al*., 2022). Environment‐specific and average yield (BLUE) were obtained from the e!DAL‐PGP‐Repository (doi: 10.5447/ipk/2022/18). Genotype‐by‐environment (GxE) interactions for grain yield were analyzed using the Additive Main Effects and Multiplicative Interaction (AMMI) model, implemented in Genstat (VSN International, Hemel Hempstead, UK), following the approach described by Malosetti *et al*. (2013). The AMMI model first applies analysis of variance (ANOVA) to partition the total variance into genotypic, environmental, and GxE interaction components. Then, principal component analysis (PCA) is applied to the GxE interaction term, extracting interaction principal components (IPCs) that summarize the interaction structure. The model is expressed as:

(Eqn 3) $$\;Y_{\mathrm{ij}}=\mu +G_{i}+E_{j}+\sum_{k=1}^{k}\alpha_{\mathrm{ik}}\gamma_{\mathrm{jk}}+\epsilon_{\mathrm{ij}}$$

where $Y_{\mathrm{ij}}$ represents the observed response of genotype i in environment j, $G_{i}$ and $E_{j}$ are the additive main effects of genotype and environment, respectively, and the interaction term is decomposed into *K* significant IPCs. Each IPC consists of a genotypic sensitivity score $\alpha_{\mathrm{ik}}$ and an environmental score $\gamma_{\mathrm{jk}}$. The IPC scores for genotypes and environments were visualized using biplots and utilized for subsequent analyses. Environmental data of temperature and precipitation at trial sites were obtained from a previous study (Zanke *et al*., 2014).

### Heritability estimation

Phenotypic data for the allometric exponent recorded in the glasshouse were merged with genome‐wide single‐nucleotide polymorphism (SNP) marker data. HapMap genotypes were converted to additive dosage scores (0/1/2), and missing values were imputed using the mean allele dosage. SNPs with a minor allele frequency (MAF) < 0.01 or a missing rate > 20% were removed. The genomic relationship matrix (GRM) was computed using the A.mat function in the rrBLUP package (Endelman, 2011). Narrow‐sense heritability (*h* ^2^) was estimated using a linear mixed model implemented in the sommer package (Covarrubias‐Pazaran, 2016). The model included developmental stage as a fixed effect and the genomic additive effect (*g*), modeled using the GRM, as a random effect. Variance components for the additive genetic effect (σ*g* ^2^) and the residual error (σ*e* ^2^) were extracted from the fitted model. Heritability was calculated as: *h* ^2^ = σ*g* ^2^/(σ*g* ^2^ + σ*e* ^2^).

### Association mapping

Genotypic data from the 35 K and 90 K iSELECT SNP arrays, along with marker oligo sequences, were obtained from the Dryad repository (Gogna *et al*., 2022). Markers were mapped to the Chinese Spring reference genome (v.1.1) using Blastn, retaining only the genomic positions with the smallest *E*‐values. Markers that could not be unambiguously positioned on the genome were excluded from the GWAS analysis. Genotypic data for functional gene markers at the *Ppd‐D1*, *Rht‐B1*, *Rht‐D1*, and *TaGW2* loci, previously reported (Zanke *et al*., 2015), were also included in the analysis. After filtering out SNPs with a MAF < 0.05, a total of 10 781 biallelic markers were used for the GWAS.

Marker–trait associations were analyzed using the R package gapit3 (Wang & Zhang, 2021) (V3.4.0) with two multilocus models: farmcpu (Liu *et al*., 2016) and blink (Huang *et al*., 2018). Population structure was assessed using PCA (PC1–PC5) of the genotypic data. Of these, the first three principal components were used as covariate fixed effects in the GWAS models to account for population structure. Markers with a false discovery rate (FDR)‐adjusted (B&H) (Benjamini & Hochberg, 1995) *P*‐value < 0.05 were considered statistically significant.

### Transcriptome analysis

The spikelet transcriptome of the photoperiod‐sensitive hexaploid wheat cultivar ‘Paragon’ was compared with two near‐isogenic lines developed by backcrossing the photoperiod‐insensitive alleles *Ppd‐A1a* (from GS‐100), *Ppd‐B1a* (Sonora64), and *Ppd‐D1a* (Sonora64) into ‘Paragon’ as described in Shaw *et al*. (2012); and another knockout line carrying frame‐shift mutations in the A copy (*Ppd‐A1_delCN*) and D copy (*Ppd‐D1_delN*), together with a deletion of the B copy (Ppd‐B1_319C), that were backcrossed to ‘Paragon’ as previously described (Shaw *et al*., 2013). RNA was extracted using Trizol (Invitrogen, USA) from central spikelets of the main spike (glumes removed) using five replicates, with each replicate consisting of spikelets pooled from at least three plants. The sequencing raw reads were first subjected to adapter trimming and quality control filtering using Fastp (Chen *et al*., 2018) (v.0.20.0) with standard parameters and workflows. We used Chinese Spring gene annotation V1.1 as a reference to estimate read abundance using the software Kallisto (Bray *et al*., 2016). Gene‐level abundance (transcripts per million, TPM) and counts were summarized with tximport (Soneson *et al*., 2015) (v3.14) DESeq2 tool (Love *et al*., 2014) was used for differentially expressed genes (DEG) analysis using genes with read counts ≥ 10 in at least three samples. A pairwise comparison was used to determine DEG by considering genotypic effect across stages (18 comparisons). Genes with |log_2_ FC| ≥ 1 and a Benjamini–Hochberg FDR‐adjusted *P*‐value < 0.05 between genotypes were considered as DEGs, resulting in total 19 022 dynamically expressed genes (DYGs). Averaged TPMs from the DYGs were Z‐scored and clustered using the K‐medoids method with the partitioning around medoids algorithm implemented in the R package cluster (Maechler, 2019) (2.1.7). The number of partitions to be clustered was set at 10 clusters based on Gap‐Statistics. Euclidean distance was used for the clustering. Enrichment analysis of Gene Ontology (GO) terms (biological processes) was conducted using the closest *Arabidopsis thaliana* homologs. The triticeae gene tribe database (Chen *et al*., 2020) was employed to identify one reciprocal best hit *Arabidopsis* homolog per wheat gene ID. GO term enrichment analysis was performed and summarized using Metascape (Zhou *et al*., 2019) with default parameters.

## Results

### Size differences contribute to variation in growth allometry

We examined growth allometry during the ontogeny of 195 winter wheat cultivars by studying the relationship between plant biomass and absolute growth rate (biomass accumulated over thermal time), which is expected to be proportional to metabolic rate (Niklas & Enquist, 2001). We utilized phenotypic data recorded during the spike growth period (Guo & Schnurbusch, 2015) reported by Guo *et al*. (2017, 2018a,b). Growth analysis began at the TS stage, which marks the cessation of spikelet primordia initiation and the onset of floret initiation. The development of floret primordia progresses through the white anther (WA) stage, reaching its maximum number by the GA stage. Following this, floret degeneration initiates around the yellow anther (YA) stage and can persist through the TP and HD stages, continuing up to AN (Supporting Information Fig. S1).

**Fig. 1:**
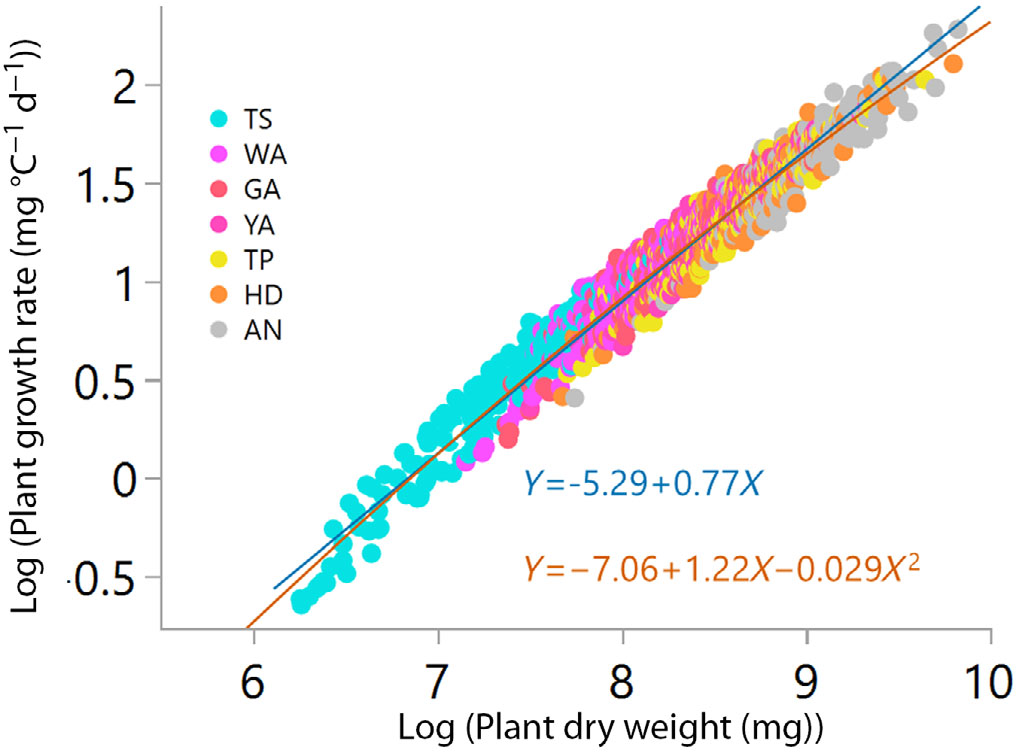
Variation in growth allometry in 195 hexaploid elite European winter wheat (*Triticum aestivum* L.) cultivars from Germany, France, Denmark, and UK grown under controlled glasshouse conditions. The blue line is the linear standard major axis regression and the orange curve is the quadratic model of growth rate allometry across developmental stages. The data represent the mean (*n* = 3) of the cultivars at each developmental stage: terminal spikelet, white anther, green anther, yellow anther, tipping, heading, and anthesis.

We fitted a linear regression across developmental stages and cultivars, determining the allometric exponent β as the slope of the allometric function *y* = α + β*X*, where *X* and *Y* represent the logarithms of plant biomass and growth rate, respectively. The allometric exponent was only slightly higher than predicted by MST and observed across land plants (β = 0.77 (CI = 0.76, 0.78)), suggesting that wheat breeding did not change growth scaling dramatically. However, a quadratic model (Akaike information criterion (AIC) = −2438) explained ontogenetic allometry better than linear regression (AIC = −2404) (Fig. 1; Table S1), suggesting that variation in allometry associates with plant size and the developmental stage of the plant. We employed the quadratic model *Y* = −7.06 + 1.22*X* − 0.029*X* ^2^ across cultivars to determine the allometric exponent of each cultivar by estimating the first derivative (β = 1.229–0.0582*X*). The allometric exponent from the quadratic function gradually decreased with ontogeny, from TS (β mean = 0.80 ± 0.02) to AN (β mean = 0.70 ± 0.02), and the cultivar‐specific allometric exponents were used in subsequent analyses.

### Variation in growth allometry is associated with resource use and reproductive strategies

**Fig. 2:**
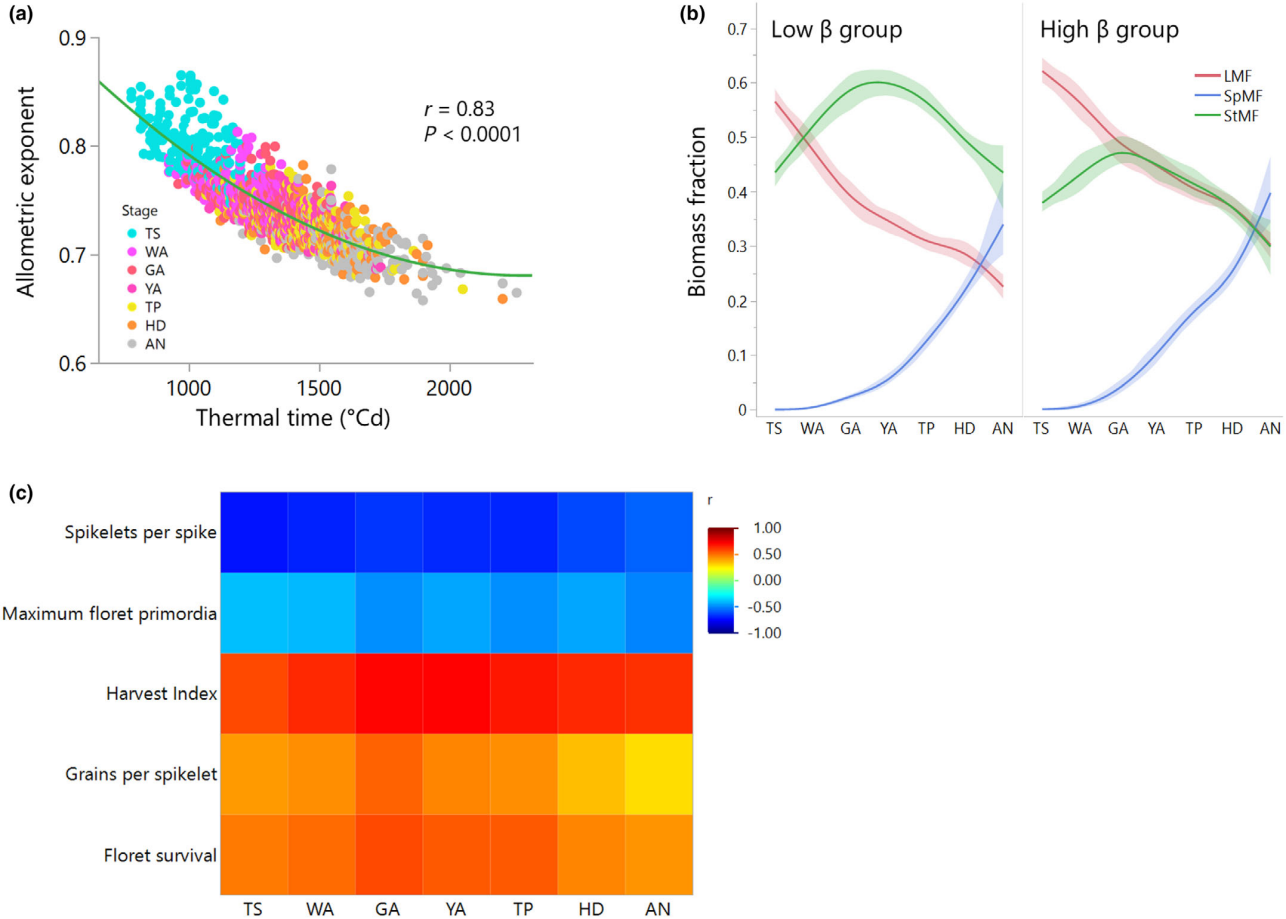
Growth allometry is associated with growth duration and reproductive traits. (a) Correlation between the allometric exponent and thermal time. (b) Biomass allocation patterns of cultivars (*n* = 5) with high (quantiles 75–90) and low (quantiles 10–25) allometric exponents (β) at green anther (GA), modeled using a spline function with a 95% confidence interval. The spline interpolates smoothly across developmental stages, providing a visualization of nonlinear trends during ontogeny. Biomass allocation is represented using mass fractions of the leaves (LMF), the stem (StMF), and the spike (SpMF). (c) Correlations of reproductive traits measured at maturity and the maximum floret primordia number measured at GA with the cultivar's growth rate allometric exponent at seven developmental stages: terminal spikelet, white anther, green anther, yellow anther, tipping, heading, and anthesis. Data from 195 hexaploid elite European winter wheat (*Triticum aestivum* L.) cultivars grown under controlled glasshouse conditions. LMF, leaf mass fraction; SpMF, spike mass fraction; StMF, stem mass fraction.

We then explored how variation in growth allometry relates to wheat growth and resource‐use strategies throughout ontogeny. To do so, we examined its association with (1) growth duration (measured as the thermal time required to reach specific developmental stages), (2) the cultivar's RGR, and (3) biomass allocation to organs. Shorter growth durations were associated with higher allometric exponents, but the magnitude of this effect declined as development progressed (Fig. 2a). Moreover, the residuals from the quadratic function correlated negatively with thermal time, which may indicate that developmental rate contributes to growth rate beyond the effects expected from plant size (Fig. S2a,b). Consistent with the diminishing returns of growth with increasing plant mass, RGR declined as plants developed (Fig. S3a), and the cultivar's allometric exponents were positively correlated with the average RGR when modeled across the entire growth phase (Fig. S3b).

Biomass allocation to the leaves decreased during ontogeny (Fig. 2b) and became closely associated with the allometric exponent, starting from the WA stage, when substantial stem and spike growth commenced (Fig. S4). Allocation to the stem increased during the floret initiation period (Fig. 2b) and peaked at the GA stage when the number of primordia per spikelet peaked (Guo & Schnurbusch, 2015). Then, allocation shifted toward reproduction, and stem growth leveled off. Comparisons between cultivars across the allometric spectrum showed that low‐exponent (larger) genotypes invested relatively more biomass in stems, whereas high‐exponent (smaller) genotypes invested more in leaves and spikes (Fig. 2b; Table S2). These allocation patterns were associated with reproductive traits: Small cultivars with higher exponents and reproductive allocation showed higher floret survival and harvest index, whereas large cultivars, which had longer reproductive phases, produced more spikelets and florets, thereby increasing their potential grain number (Fig. S5).

Previous studies have highlighted the role of growth duration during the critical period of stem elongation in determining spike fertility (Miralles *et al*., 2000; González *et al*., 2003; Prieto *et al*., 2018). We therefore tested whether the observed correlations between the allometric exponent and reproductive traits could be similarly associated with variation in phenology. Analyses of residuals from regressions of reproductive traits against thermal time revealed that the allometric exponent still accounted for 10–30% of the variation in reproductive traits (Fig. S6), indicating that it captures biological information beyond phenology. Although growth allometry was associated with reproductive development across stages, the strength of these correlations varied (Fig. 2c), indicating that the effect of plant size on reproductive traits in wheat depends on the developmental stage. Overall, our results suggest that variation in growth allometry may reflect selection for resource use and reproductive strategies, and highlight the importance of plant size in determining yield at specific developmental windows.

### Genetic variation in growth allometry is associated with genotype–environment interactions for yield in independent field trials

**Fig. 3:**
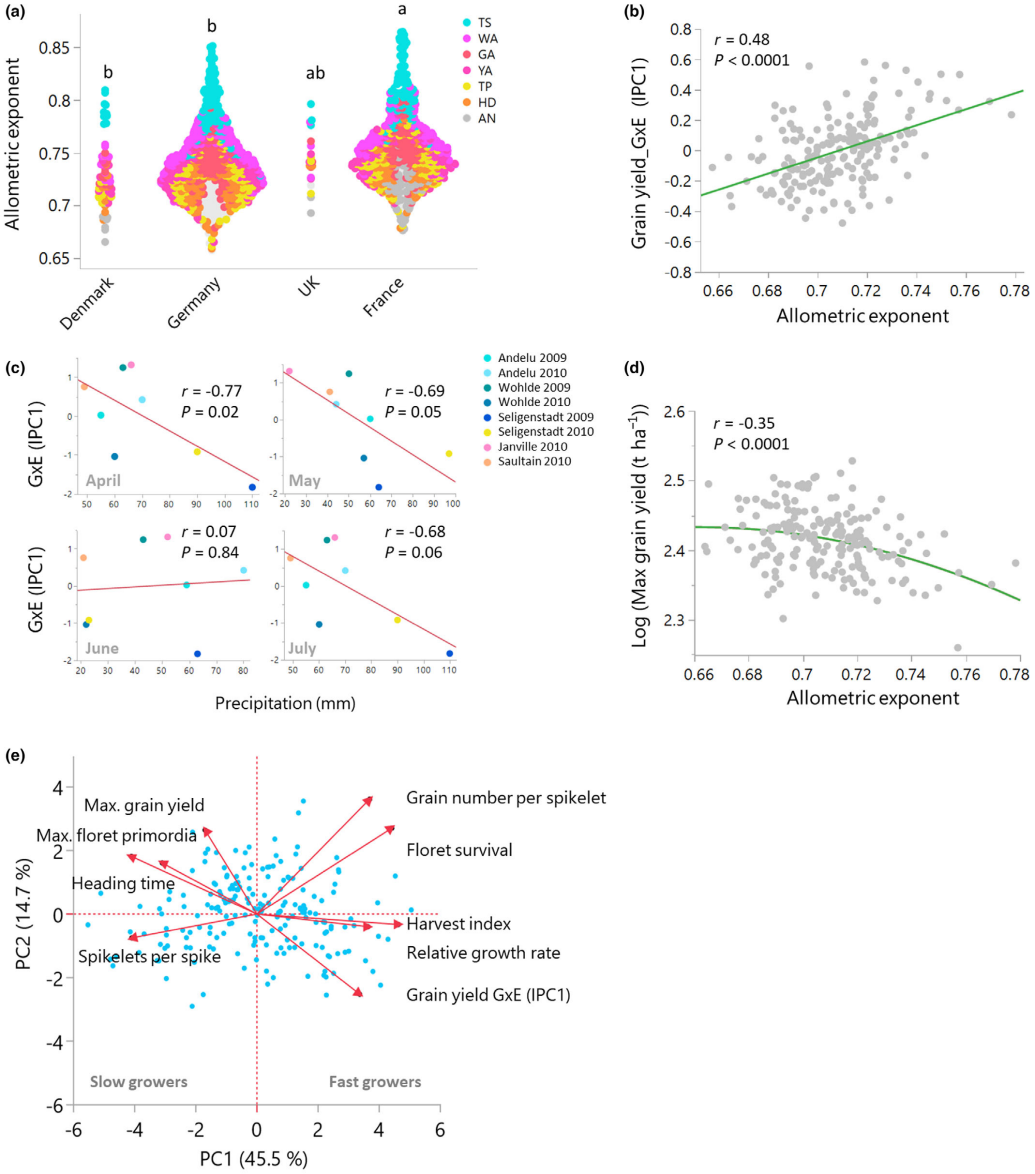
Growth rate allometry is associated with environmental adaptation in 195 hexaploid elite European winter wheat (*Triticum aestivum* L.) cultivars from Germany, France, Denmark, and the UK. (a) Associations between the cultivar's country of registration and the allometric exponent at different developmental stages. Letters indicate statistically significant differences as determined by Tukey–Kramer honestly significant difference (*P* < 0.05). (b) Correlation between the allometric exponent at anthesis in glasshouse‐grown plants and genotype‐by‐environment interactions for yield in rain‐fed field trials in Germany and France. (c) Correlation between the environment IPC1 and the monthly precipitation levels at the field sites. (d) Correlation between the allometric exponent at anthesis and the highest grain yield recorded across eight different environments. (e) PCA illustrating the differentiation of adaptive strategies shaped by growth allometry within the phenotypic space. IPC, interaction principal component; PCA, principal component analysis.

Having found close relationships between reproductive traits and growth allometry, we examined whether growth allometry correlates with grain yield under agronomically realistic conditions. This is biologically important and relevant in the context of plant breeding in which phenotyping in simplified platforms, such as glasshouse or growth chambers, usually returns traits that do not correlate with grain yield in the field (Sadras, 2019). To address this, we investigated grain yield data of the same cultivars grown in eight distinct field environments (Table S3), resulting from the combination of seasons and sites in Germany and France (Gogna *et al*., 2022). Averaged yield varied from 7.9 t/ha to 11 t/ha across environments and from 8.5 t/ha to 10.6 t/ha across cultivars. The cultivar's average grain yield across environments best linear unbiased estimator (BLUE) was unrelated to the allometric exponent and showed no trend with the cultivar's year of registration (Fig S7a,b). However, growth allometry was related to the country of origin of the cultivar. ANOVA indicated that the country of origin of the cultivar accounted for up to 23% (*P* < 0.0001) of the variation in the allometric exponents (Table S4). French cultivars have allometric exponents significantly higher than Danish and German cultivars (Fig. 3a), suggesting that breeding for grain yield in different environments has favored adaptive patterns of growth allometry and developmental timing.

Thus, we tested whether the variation in growth allometry is related to the cultivar's performance in contrasting environments. To address this, we used the AMMI model (Zobel *et al*., 1988) to dissect variation in GxE interactions into principal components for both genotypes and environments. This enabled us to investigate the associations between G×E interactions and the cultivar's allometric exponent, as well as between G×E and weather conditions during the field trials. IPC1 explained 31.7% of the G×E interactions and IPC2 accounted for 19.4% of the variation (Fig. S8). The allometric exponent recorded under controlled conditions explained 23% of the variation of the genotype IPC1 from independent field trials (Fig. 3b). Genotypes with higher allometric exponents performed relatively better in certain environments (2010Janville, 2009Wohlde), positioned farther from the origin in the AMMI biplot, indicating stronger genotype–environment interactions.

We then examined the relationship between the environmental IPC, which reflects the interactive forces of the environment, and the monthly rainfall and average temperatures at the trial sites. Environmental IPC1 scores were unrelated to monthly average temperatures (Fig. S9) and correlated with precipitation (Fig. 3c), highlighting the role of precipitation in shaping GxE interactions of rain‐fed crops. Considering also genotype‐dependent IPC1 correlations with the allometric exponents (Fig. 3b), we conclude that cultivars with high allometric exponents are better adapted to environments with lower rainfall during stem elongation (April–May) and grain filling (July), although this association may partly reflect adaptive variation in phenology that covaries with allometry. Taken together, genotype‐dependent growth allometry is important for environment‐specific cultivar performance in major European wheat producing countries.

Notably, the allometric exponent was correlated with the cultivar's maximum grain yield across environments, with the strongest correlation observed when the exponent was estimated at AN. Cultivars with lower allometric exponents, associated with a higher number of spikelets and floret primordia, exhibited higher maximum yield. The observed relationship indicates actual yield associates with growth allometry (Fig. 3d). Although the scatter of yield is typical of crops grown in realistic field conditions, the consistency and significance across different analyses suggest that growth allometry captures meaningful adaptive strategies. Overall, cultivars were distributed along a trade‐off of reproductive strategies. On the one side, fast‐growing, smaller genotypes produce fewer spikelets per spike but maintain a higher proportion of viable florets per spikelet, return a higher harvest index, and yield well under low rainfall. On the other side, larger cultivars that prioritize the initiation of spikelet and floret primordia achieve a higher maximum yield despite potentially lower floret survival (Fig. 3e).

### The genetic and molecular basis of growth allometry are linked to G×E interactions for grain yield in the field

To gain deeper insights into the genetics of the plant‐ and stand‐level traits investigated, we conducted a GWA scan with a focus on genomic regions associated with allometry, organ allocation patterns, growth rate, and growth duration measured in the glasshouse, and the G×E interactions for yield quantified in the field. We utilized the publicly available genotypic information of the cultivars (Gogna *et al*., 2022) to analyze the population structure. PCA showed that PC1 explained 8.8% of the genetic variation and PC2 accounted for 4.3%. Although no clear distinction of subpopulations was observed within the panel, German cultivars displayed lower average PC1 values than French and Danish cultivars, suggesting genetic differentiation by country of registration (Fig. S10a,b). The allometric exponent was weakly correlated with PC1, PC3, and PC5 (Fig. S10c).

**Fig. 4:**
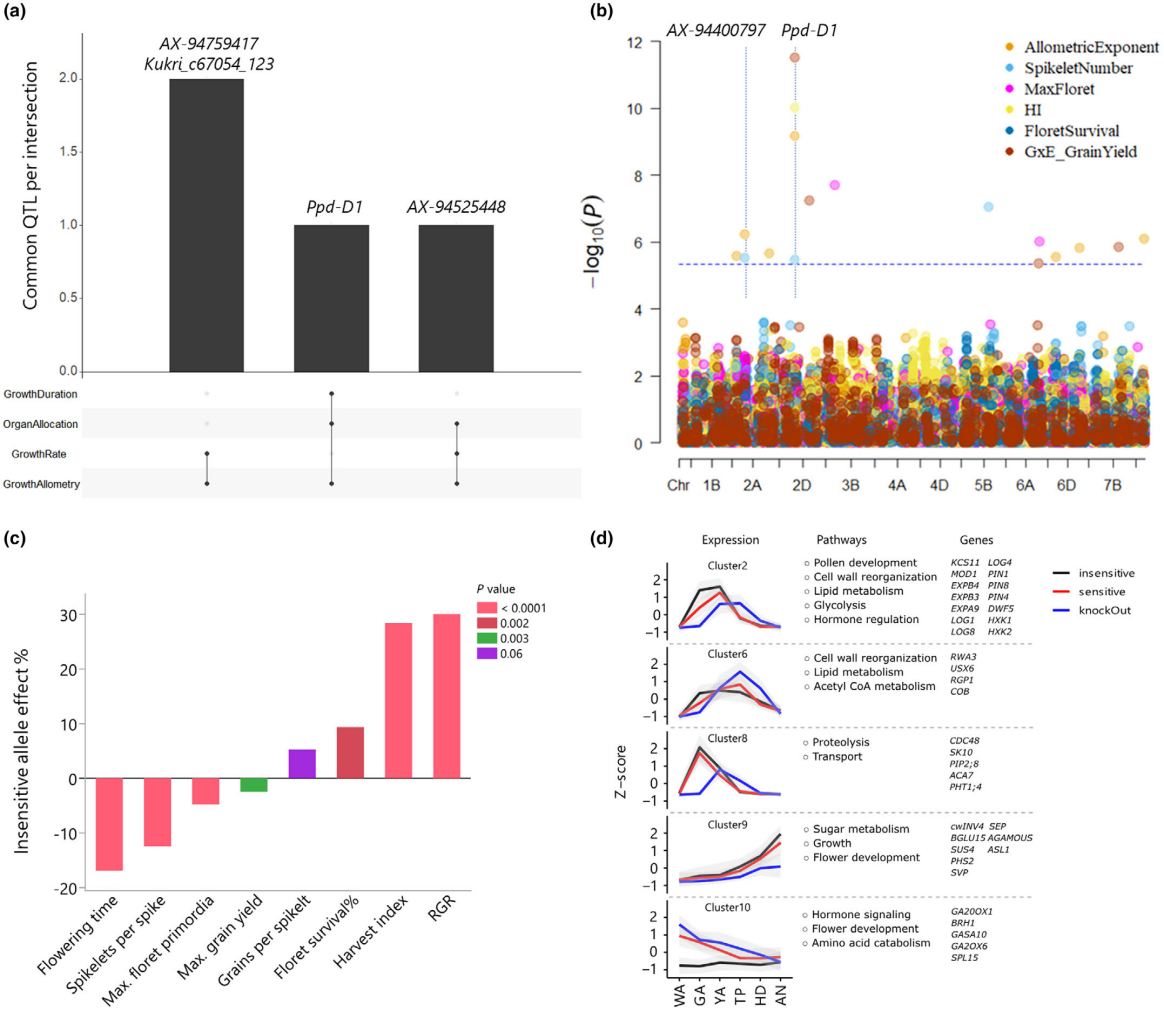
Genetic and molecular basis of growth rate allometry in 195 hexaploid elite European winter wheat (*Triticum aestivum* L.) cultivars. (a) UpSet plot depicting common QTL for allometry with growth traits. (b) Multitrait Manhattan plot demonstrating shared genetic basis for growth allometry, reproductive traits, and grain yield genotype‐by‐environment in eight environments. Horizontal dashed line indicates the Bonferroni correction threshold. Vertical dashed lines indicate common marker–trait associations. (c) The effects of the *Photoperiod‐D1* (*Ppd‐D1*) insensitive allele on reproductive traits and relative growth rate. (d) *Ppd‐1* allele driven spikelet gene expression dynamics from white anther to anthesis. Gene clusters along with representative pathways and genes from enrichment analysis.

The allometric exponent showed high narrow‐sense heritability (0.75–0.85 across developmental stages; Table S5), suggesting that variation in allometry is largely genetically controlled. Our GWAS analysis identified 36 significant marker–trait associations (FDR < 0.05) with growth allometry, corresponding to 19 quantitative trait locus (QTL) (Table S6). Of these, four QTL were also associated with organ allocation, growth rate, and duration, indicating a common genetic basis for growth allometry and resource‐use strategies (Fig. 4a). One of the most prominent variants associated with growth allometry was a *c*. 2 kilo base pair (kb) deletion upstream of the *Photoperiod 1* gene (*Ppd‐D1*) (Beales *et al*., 2007). This deletion showed a consistent signal across multiple traits and developmental stages (Table S6) and was present in 15% of the tested cultivars, all of them registered in France, highlighting a potential mechanistic link between developmental timing, biomass allocation, and allometric scaling, and motivating our focus on *Ppd‐1*. This variant confers photoperiod insensitivity, resulting in a higher developmental rate and shorter growth periods. Further GWAS and pairwise comparisons of different *Ppd‐D1* alleles showed that this allelic variation influences key reproductive traits, such as spikelet number and harvest index, and is associated with RGR, maximum floret primordia, and floret survival (Table S6; Fig. 4b,c). The photoperiod‐sensitive allele associated with spikelet and floret initiation, while the insensitive allele increased floret survival and harvest index at maturity. These associations may reflect, in part, the primary role of phenology in shaping growth allometry and environmental adaptation. A previous study of *Ppd‐D1* effects on reproductive development using near‐isogenic lines and a loss of function mutant corroborated gene effects on spikelet number and fertility (Liu *et al*., 2020). Notably, *Ppd‐D1*, which associated with resource allocation, growth duration, and reproductive strategies, also associated with G×E interactions for grain yield captured in principal components (IPC1) derived from the AMMI model (Fig. 4b; Table S6). This provides a genetic substrate to the phenotypic association between allometry traits and yield in the field.

To investigate how scaling of metabolism and growth are reflected at the molecular level and explore links with reproductive growth, we reanalyzed the spikelet transcriptome of three Paragon‐derived genotypes with differing *Ppd‐1* alleles across six developmental stages from WA to AN (Liu *et al*., 2020). Pairwise comparisons among genotypes identified *c*. 19 000 DEGs, grouped into 10 clusters using the K‐Medoids algorithm (Fig. S11; Table S7). Five clusters exhibited distinct allele effects on gene expression timing and intensity, driven primarily by heterochronic shifts (Fig. 4d). For example, cluster C2 showed early and higher expression in the photoperiod‐insensitive genotype, peaking at GA, at the peak of floret primordia formation, and declining after YA. This cluster was enriched in genes related to cell wall modification (e.g. expansins), glycolysis (e.g. hexokinase 1, hexokinase 2), lipid metabolism (e.g. mosaic death 1, 3‐ketoacyl‐CoA synthase 11), and cytokinin and brassinosteroid biosynthesis (e.g. lonely guys, dwarf 5), reflecting increased metabolic investments that accelerate early spike growth. Similarly, cluster C6 displayed early induction of genes involved in cell wall organization, lipid biosynthesis, acetyl‐CoA metabolism, and transmembrane transport in the insensitive genotype.

Cluster C8 showed specific expression of protein catabolism and membrane transport genes at GA in wild type (WT) and near isogenic lines but delayed in the KO. Genes in cluster C9, associated with sugar metabolism (e.g. cell wall invertase 4, sucrose synthase 4) and floral development (e.g. sepallata 3, short vegetative phase), exhibited the highest expression in the insensitive genotype and the lowest in KO. These patterns highlight the role of *Ppd‐1* in metabolic scaling, enabling faster growth. By contrast, cluster C10 showed consistently low expression in the insensitive line, whereas WT and KO exhibited declining expression patterns. This cluster was enriched with gibberellin‐related genes, potentially influenced by the high expression of cytokinin biosynthesis genes in the insensitive line during early spike growth. Considering the spikelet transcriptome alongside the performance of *Ppd‐D1* across environments, we propose that the early metabolic upregulation observed in *Ppd‐D1* insensitive genotypes represents an adaptive strategy for efficient resource utilization in environments where rapid reproductive development is adaptive, particularly under water deficit.

## Discussion

The metabolic and growth rates of plants influence their resource requirements (Brown *et al*., 2004). Understanding how growth rate changes throughout plant development is crucial for determining how resource use relates to growth patterns, reproductive development, and performance in different environments, informing fertilization practices, improving modeling of cultivar responses to environmental conditions, cultivar selection, and enhancing crop growth and yield prediction (Lemaire *et al*., 2019; Weiner *et al*., 2021; Westgeest *et al*., 2024).

Our large‐scale dataset, encompassing high‐resolution ontogenetic dynamics paired with grain yield data from eight contrasting field environments (Table S8), revealed developmental, physiological, genetic, and molecular pathways linking individual plant growth allometry under controlled conditions to grain yield in the field. By integrating ecological theory with crop science, we uncover how resource‐use strategies covary with reproductive development, and show how patterns of growth allometry correspond to variation in grain yield. Our findings demonstrate that size–trait relationships linked to developmental rate are prevalent and underlie key agronomic traits, and that artificial selection has been accompanied by shifts in growth and metabolic scaling associated with environmental adaptation. These results highlight the potential of the allometric framework to improve understanding, prediction, and manipulation of crop performance.

The nonlinear relationship between size and growth rate reflects variation in short‐term growth dynamics among cultivars, variation that is averaged out in a linear regression across a 35‐fold range of plant mass (Fig. 1). While likely not a direct target of selection, this variation is highly relevant for crop adaptation. Moreover, the robustness of allometry, that is scaling from controlled conditions to the field, opens new opportunities for screening breeding material for allometric traits at the individual‐plant level to enhance genetic gain at the stand level (Sadras, 2019; Golan *et al*., 2023).

Indeed, we found that breeding in contrasting environments has been accompanied by shifts in growth allometry, reflecting correlated changes in developmental rate and organ allocation that influence yield components such as floret survival and harvest index, while compromising on potential grain number. Even though crop improvement partially relies on empirical breeding and selection, we did not observe time trends in growth allometry during the contemporary breeding of European cultivars (Fig. S7). This may be because variation in growth allometry may result from grain yield selections in specific environments. In France, larger latitudinal variation in climate and soil likely requires more environment‐specific breeding. Small, fast‐developing plants with high allometric exponents are agronomically adapted to the southern regions, where extended dry periods are common. On the other hand, slow‐developing, large genotypes can return higher yield in wetter, higher‐yielding environments of northern Europe.

This association may at least in part reflect adaptive variation in phenology that is integrated into allometry. Breeding for yield in different climates results in phenology suited to local temperature and rainfall regimes, and faster‐developing cultivars usually perform better under dry and warm conditions. However, our analyses accounting for phenology (Fig. S6) indicate that growth allometry captures additional physiological variation, suggesting that growth allometry reflects not only developmental rate but also intrinsic differences in growth rates and resource allocation. Thus, allometry provides an integrative framework linking developmental timing with physiological strategies that determine yield across environments.

Long‐term genetic yield improvements in Europe, the United States, and Argentina have been attributed to a balanced contribution of increased yield potential and maintenance breeding, ensuring cultivars remain adapted to evolving abiotic and biotic conditions (Grassini *et al*., 2025), highlighting an untapped potential for incorporating growth allometry in breeding, for example through direct selection of allometric parameters to enhance adaptation to different environments. Interestingly, a study using the complete GABI wheat panel modeled the temperature response of stem elongation rate and found that French cultivars exhibit a more pronounced increase in elongation rate with rising temperatures than German cultivars (Roth *et al*., 2023). This response, which is likely associated with faster leaf development and radiation interception that may contribute to their fast growth, is reflected in higher allometric exponents and adaptation to low rainfall shown in our study.

Allometry can be considered a form of phenotypic integration, arising from variations in developmental processes that lead to the covariation of multiple traits across organization levels (Hallgrímsson *et al*., 2019; Vasseur *et al*., 2022). Most tellingly, our genetic and phenotypic analysis identifies *Ppd‐1* as a key regulator of vegetative and reproductive trait covariation integrated within growth allometry. *Ppd‐1* functions within the photoperiod pathway, regulating genes underlying developmental timing (Guo *et al*., 2018a; Gauley *et al*., 2024), thereby determining developmental windows for organ growth. Moreover, *Ppd‐1* directly activates the gibberellin biosynthesis pathway in internodes, thereby influencing elongation processes (Huang *et al*., 2024). Consequently, as shown here, allelic *cis*‐variation at *Ppd‐1* associates with variation in developmental timing, resource allocation, and growth rate, suggesting that *Ppd‐1* may affect adaptation not only through its effect on development but also by directly affecting growth processes.

Gradual increases of flowering signals during reproductive development modulate fertility and grain yield (Shaw *et al*., 2018; Gauley *et al*., 2024; Yoshikawa & Boden, 2024). The photoperiod‐sensitive alleles (lower signal intensity) allow longer periods of spikelet initiation, whereas the stronger flowering signal in the photoperiod‐insensitive lines elicits the early induction of metabolic and floral development genes that promote floret survival, including regulators of cytokinin and sugar levels (Fig. 4d). In line with our findings, *Ppd‐1* plays a role in stabilizing barley (*Hordeum vulgare* L.) reproductive output under high temperatures by boosting metabolic genes in the spike, allowing acceleration of reproductive development to mitigate temperature effects on floret fertility (Lan *et al*., 2025).

Growth allometry is a key integrator for not only developmental but also environmental signals. Environmental factors significantly affect plant size and consequently affect resource allocation in the plant (Weiner, 2004). Here, the interaction between plant size and the developmental stage is important. For example, resource limitation reducing plant size before the allocation to the stem reaches its maximum (Fig. 2b) will increase the proportion of leaves. Beyond this point, size reductions will affect reproductive allocation, and the proportion of vegetative parts will become larger. However, the genotype's allometric trajectory for reproductive allocation during this critical growth phase influences the degree of phenotypic plasticity in reproductive growth. A steeper increase in reproductive vs vegetative growth will promote yield but will also make the genotype more sensitive to environmental stresses (Qin *et al*., 2013; Du *et al*., 2020; Weiner *et al*., 2021).

Overall, our study bridges a gap between fundamental understanding of plant allometry and agronomic applications. Our finding that plant allometry scales to grain yield under relevant field conditions, alongside genetic and metabolic evidence, suggests – while acknowledging the influence of phenology – that an allometric perspective would be useful for crop phenotyping in a breeding context (Westgeest *et al*., 2024).

## Competing interests

None declared.

## Author contributions

GG was involved in conceptualization, data analysis and writing of the first draft. FV was involved in writing, manuscript review and editing. YH was involved in transcriptome analysis, writing, manuscript review and editing. KT was involved in bioinformatic processing of transcriptome data. VOS was involved in writing, manuscript review and editing. CV was involved in writing, manuscript review and editing. TS was involved in funding acquisition, writing, manuscript review and editing.

## Disclaimer

The New Phytologist Foundation remains neutral with regard to jurisdictional claims in maps and in any institutional affiliations.

## Supporting information


**Fig. S1** Schematic representation of the initiation and degenerations of floret primordia along the spikelet development scale defined by Kirby and Appleyard (1981).
**Fig. S2** Residuals analysis of ontogenetic allometry.
**Fig. S3** Variation in relative growth rate across development and cultivars.
**Fig. S4** Correlation between the leaf mass fraction and the allometric exponent throughout ontogeny.
**Fig. S5** Development of reproductive traits and their associations with the growth rate allometric exponent during yield‐defining stages.
**Fig. S6** Correlations between the allometric exponent and reproductive traits after accounting for variation in phenology.
**Fig. S7** Growth allometry do not affect stable grain yield effects.
**Fig. S8** Visualization of G × E interactions for grain yield across eight environments in Germany and France.
**Fig. S9** Correlations between the average temperature in the eight field trials with the interaction principal components extracted from the AMMI model.
**Fig. S10** Population structure of the GABI wheat panel and its association with the country of origin and the allometric exponent.
**Fig. S11** Gene expression in the spikelets of near‐isogenic lines with differing *Ppd‐1* alleles, observed from WA to AN.

**Table S1** Parameters of ontogenetic allometry.
**Table S2** Interspecific allometric parameters between Log (organ) and Log (whole tiller mass).
**Table S3** Environmental conditions at the field sites.
**Table S4** Analysis of variance examining the effects of the cultivar's country of registration on the allometric exponent.
**Table S5** Estimates of narrow‐sense heritability and variance components across developmental stages.
**Table S6** Significant SNP detected using Blink and FarmCPU across developmental stages.
**Table S7** List of DEG's between genotypes and their respective mean TPM values.
**Table S8** phenotypic values of 195 European winter wheat cultivars measured during ontogeny and at maturity.
Please note: Wiley is not responsible for the content or functionality of any Supporting Information supplied by the authors. Any queries (other than missing material) should be directed to the *New Phytologist* Central Office.

## Data Availability

The data that support the findings of this study are available in the Supporting Information (Tables S7 and S8) of this article.
