# Supplementary material for: Scaling law links plant growth variation to grain yield in wheat stands
Source: New Phytol. 2026 Feb 17;250(4):2163–75. doi: 10.1111/nph.71028 (PMC13103433; doi:10.1111/nph.71028)
Supplement: Supplementary file 1 — Fig. S1 Schematic representation of the initiation and degenerations of floret primordia along the spikelet development scale defined by Kirby and Appleyard (1981). Fig. S2 Residuals analysis of ontogenetic allometry. Fig. S3 Variation in relative growth rate across development and cultivars. Fig. S4 Correlation between the leaf mass fraction and the allometric exponent throughout ontogeny. Fig. S5 Development of reproductive traits and their associations with the growth rate allometric exponent during yield‐defining stages. Fig. S6 Correlations between the allometric exponent and reproductive traits after accounting for variation in phenology. Fig. S7 Growth allometry do not affect stable grain yield effects. Fig. S8 Visualization of G × E interactions for grain yield across eight environments in Germany and France. Fig. S9 Correlations between the average temperature in the eight field trials with the interaction principal components extracted from the AMMI model. Fig. S10 Population structure of the GABI wheat panel and its association with the country of origin and the allometric exponent. Fig. S11 Gene expression in the spikelets of near‐isogenic lines with differing Ppd‐1 alleles, observed from WA to AN. [file NPH-250-2163-s001.pdf]

**New Phytologist Supporting Information**

Article title:
Scaling law links plant growth variation to grain yield in wheat stands

Authors:
Guy Golan, François Vasseur, Yongyu Huang, Kenan Tan, Victor O. Sadras, Cyrille Violle, Thorsten Schnurbusch

Article acceptance date:
02 February 2026

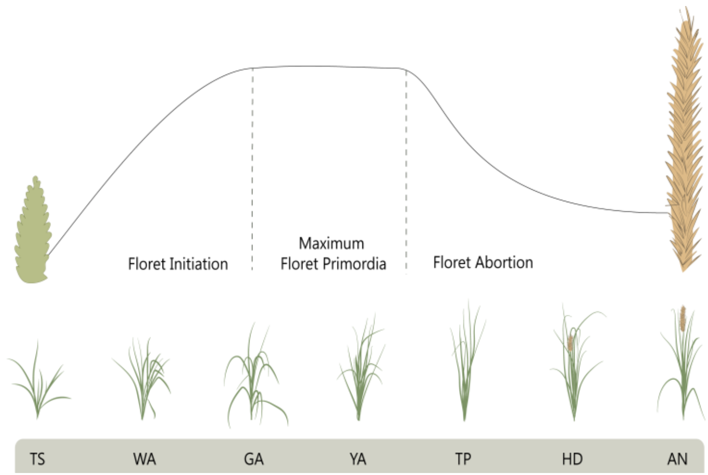


**Fig. S1** Schematic representation of the initiation and degenerations of floret primordia along the spikelet development scale defined by Kirby and Appleyard (1984).

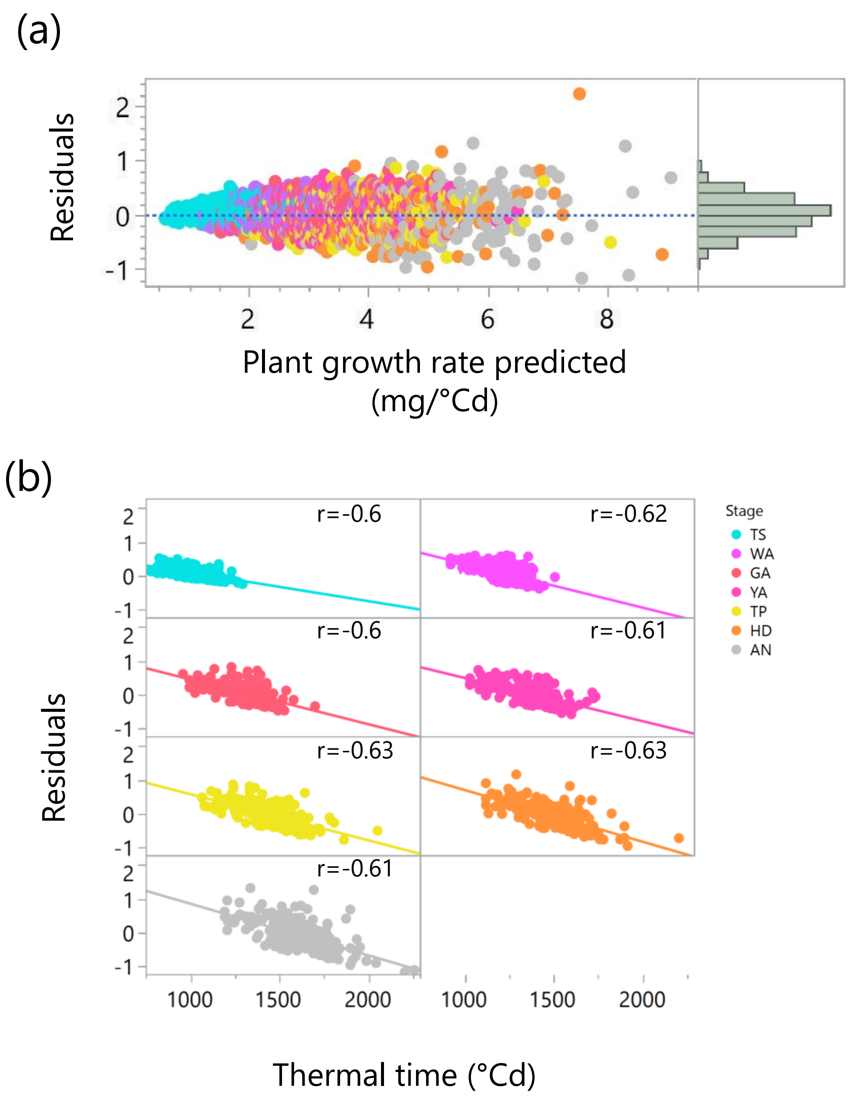


**Fig. S2** Residuals analysis of ontogenetic allometry. (a) Predicted growth rate vs residuals of the quadratic model presented in Fig. 1. (b) Correlations between growth duration and residuals. All correlation P-values were below 0.0001.

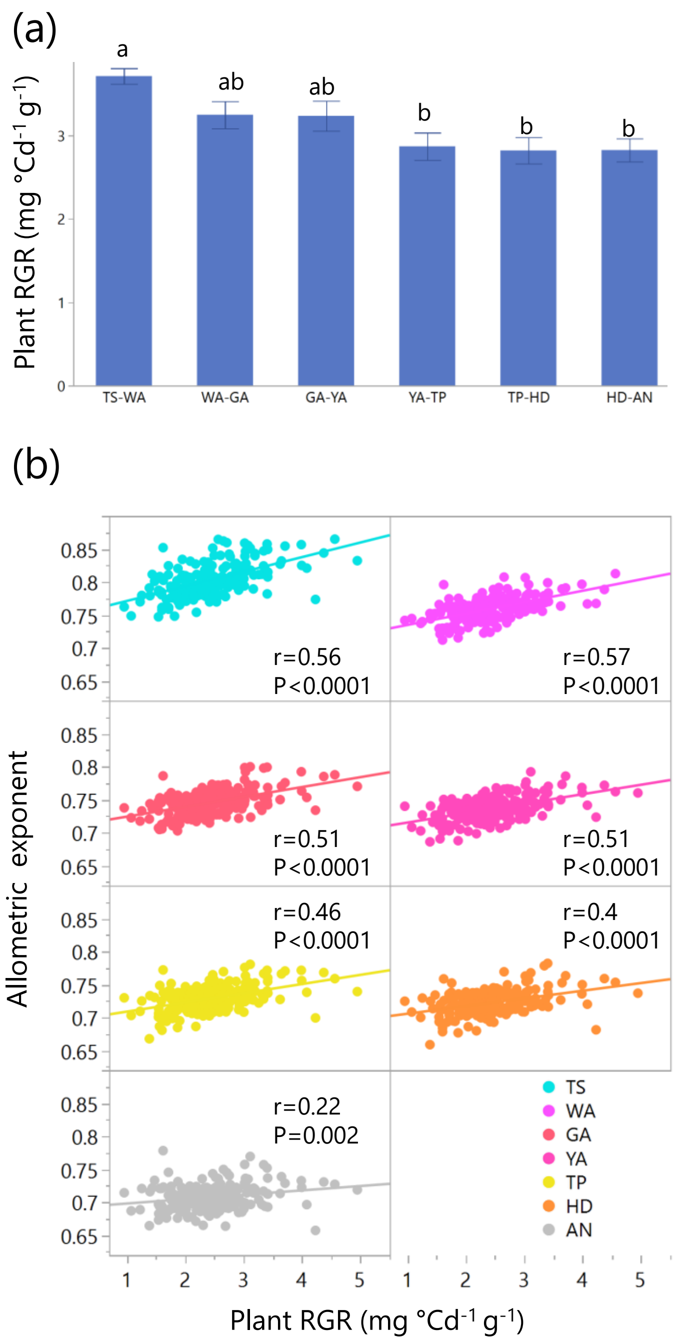


**Fig. S3** (a) Changes in RGR calculated between successive developmental stages. (b) Correlations between the cultivar's relative growth rate (RGR) modeled across stages and the allometric exponent at different developmental stages. Error bars indicate the standard error. Different letters indicate statistically significant differences (Tukey-Kramer HSD, $P<0.05$).

(a)

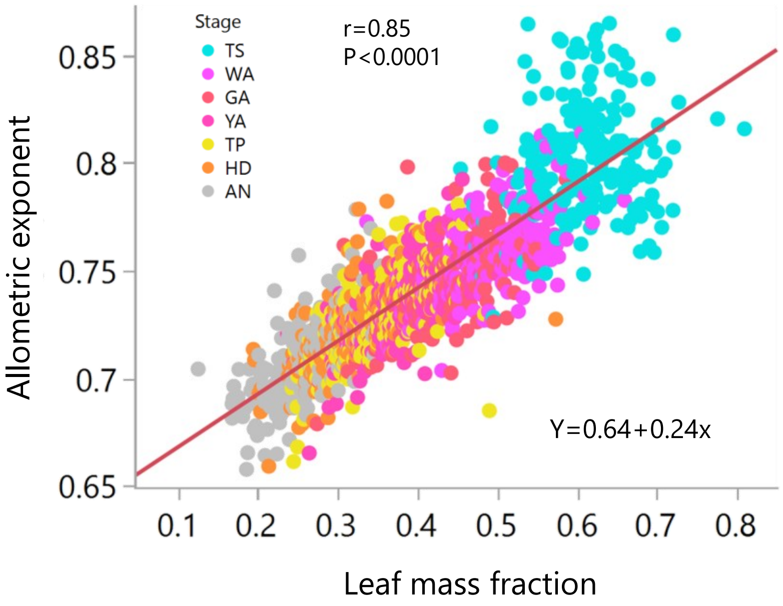


**Fig. S4** Correlation between the leaf mass fraction (LMF) and the allometric exponent throughout ontogeny.

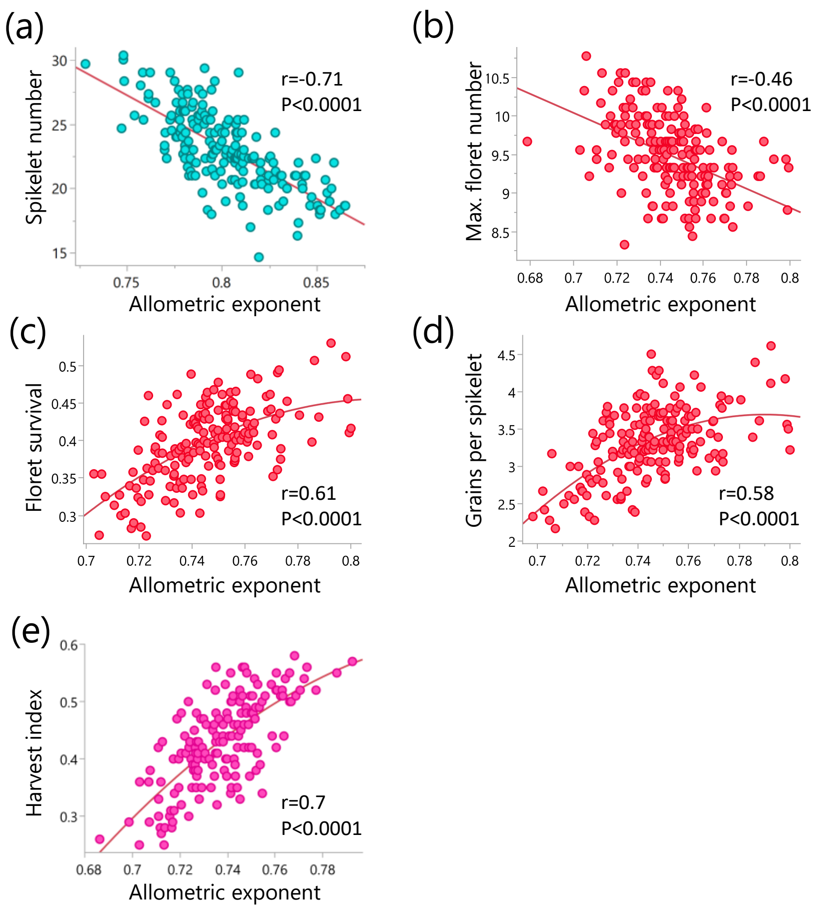


**Fig. S5** Development of reproductive traits and their associations with the growth rate allometric exponent. (a) Correlation between the allometric exponent at TS and the number of spikelets per spike. (b) Correlation between the allometric exponent at GA and the maximum number of floret primordia. (c) Correlation between the allometric exponent at GA and the percentage of floret primordia that ended up setting grains (d) Correlation between the allometric exponent at GA and the number of grains per spikelet. (e) Correlation between the allometric exponent at YA and the harvest index at maturity.

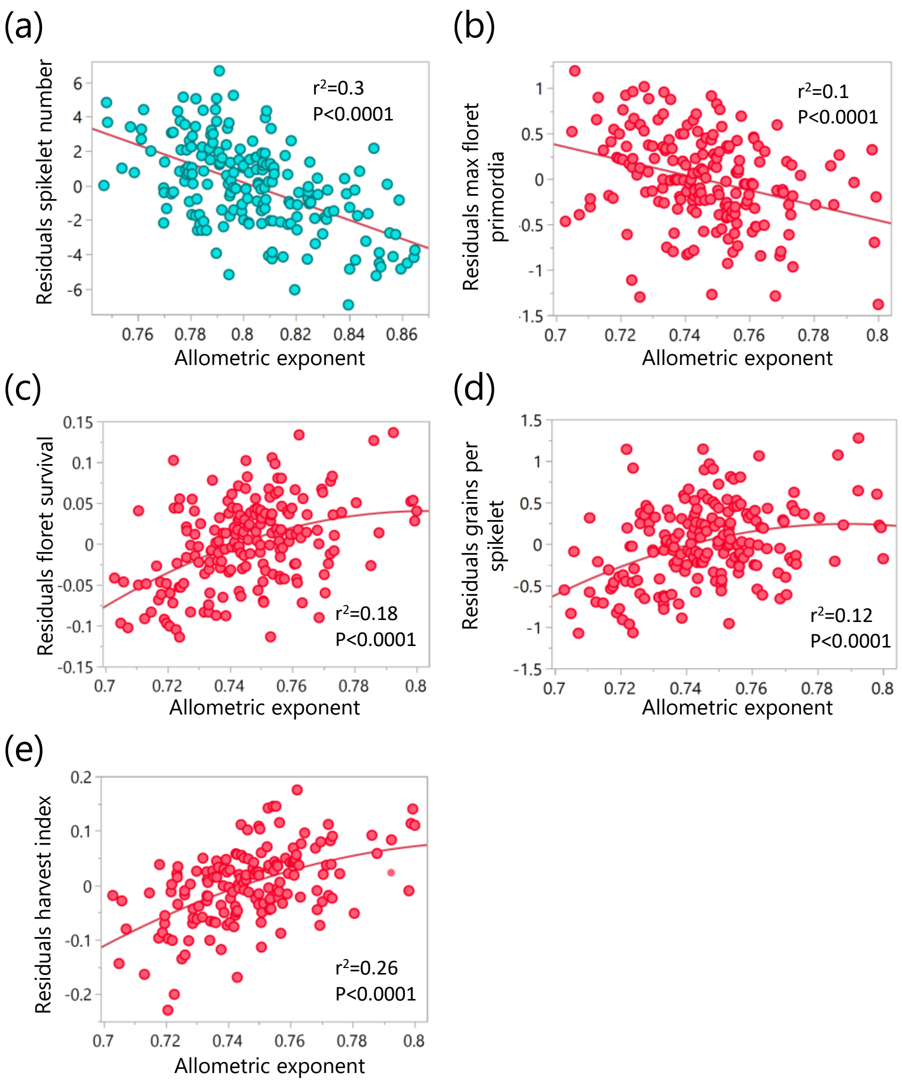


**Fig. S6** Correlations between the allometric exponent and reproductive traits after accounting for variation in phenology**.** (a) Correlation between the residuals of spikelet number against thermal time at TS and the allometric exponent. (b) Correlation between the residuals of max floret primordia against thermal time at GA and the allometric exponent. (c) Correlation between the residuals of floret survival against thermal time at GA and the allometric exponent. (d) Correlation between the residuals of grains per spikelet against thermal time at GA and the allometric exponent. (e) Correlation between the residuals of harvest index against thermal time at GA and the allometric exponent.

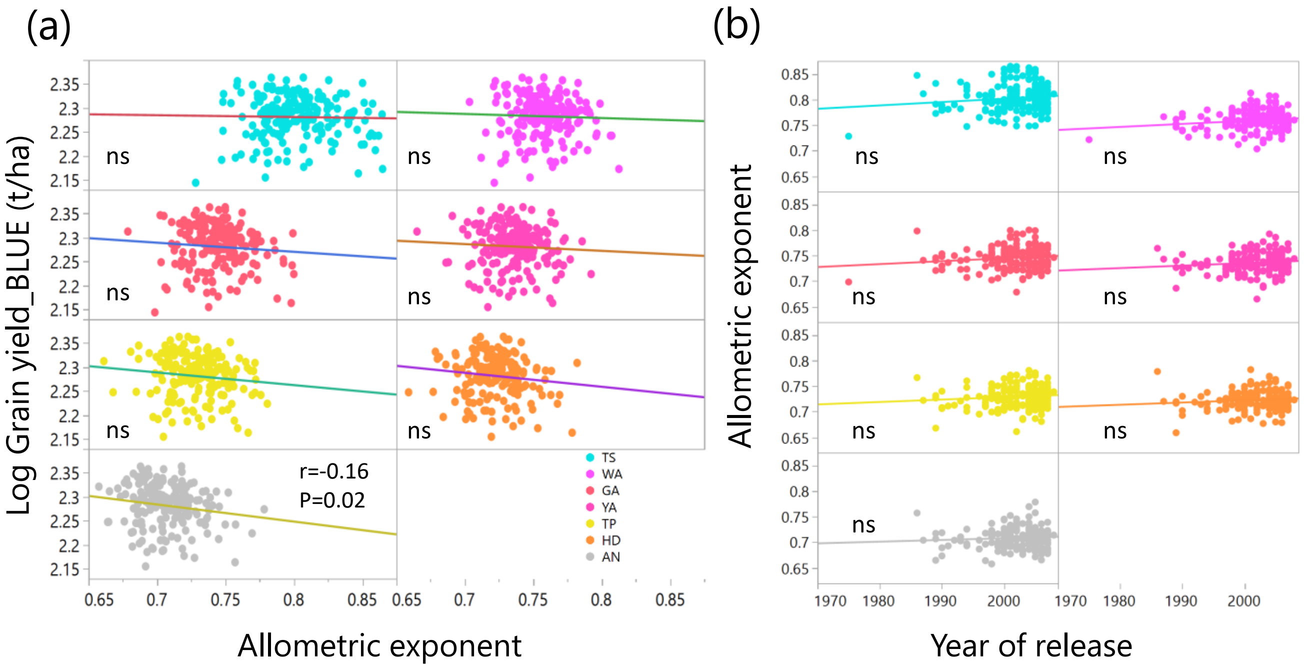


**Fig. S7** Growth allometry do not affect stable grain yield effects. (a) Relationships between the allometric exponent at different stages and the cultivar's grain yield per area, averaged across eight environments. (b) Relationships between the cultivar's year of release and the allometric exponent at different developmental stages.

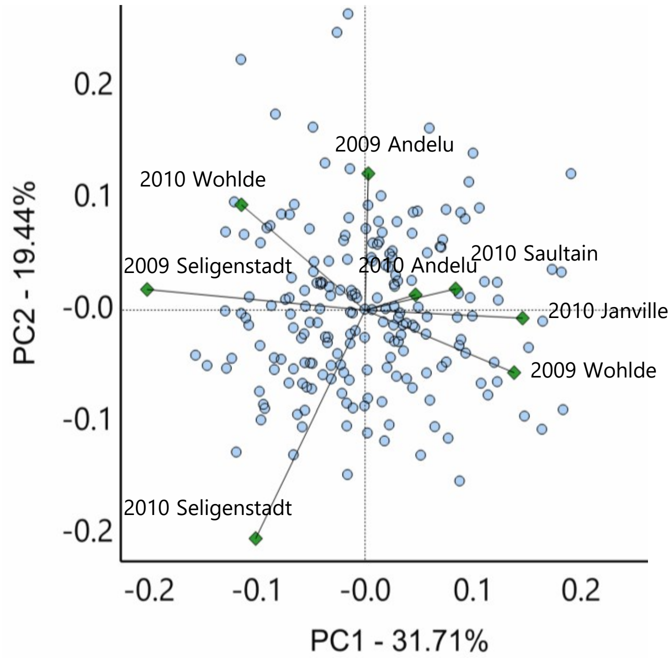


**Fig. S8** Visualization of G × E interactions for grain yield across eight environments in Germany and France. A biplot using symmetric scaling represents cultivars as blue circles and environments as green diamonds. Cultivars near the origin are less sensitive to environmental interactions, whereas those located farther from the origin exhibit specific adaptation.

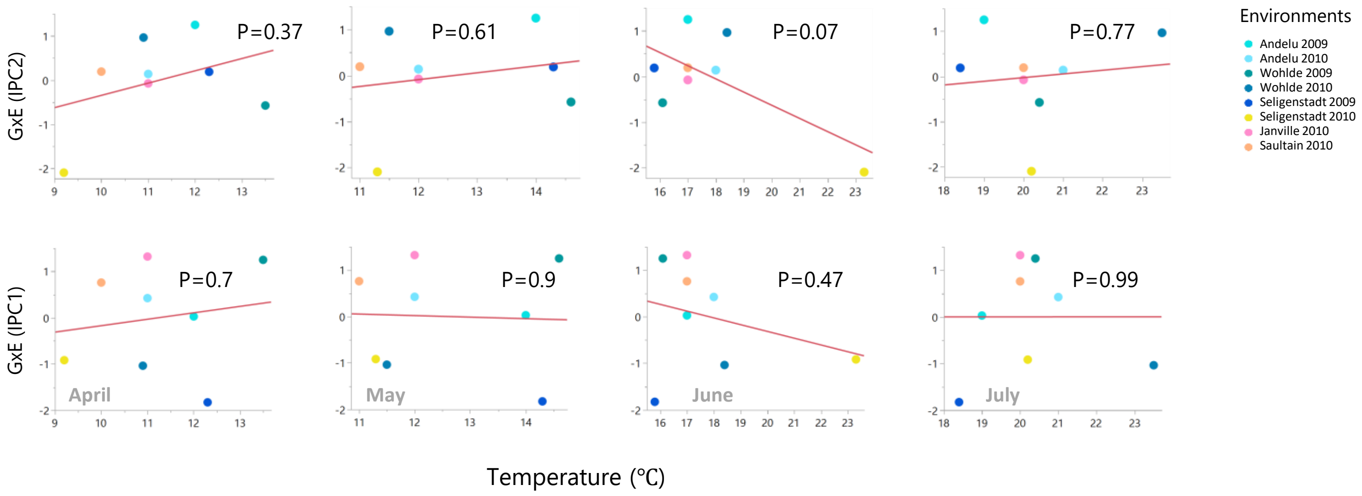


**Fig. S9** Correlations between the average temperature(Celsius degrees) in the eight field trials with the interaction principal components (IPC) extracted from the AMMI model.

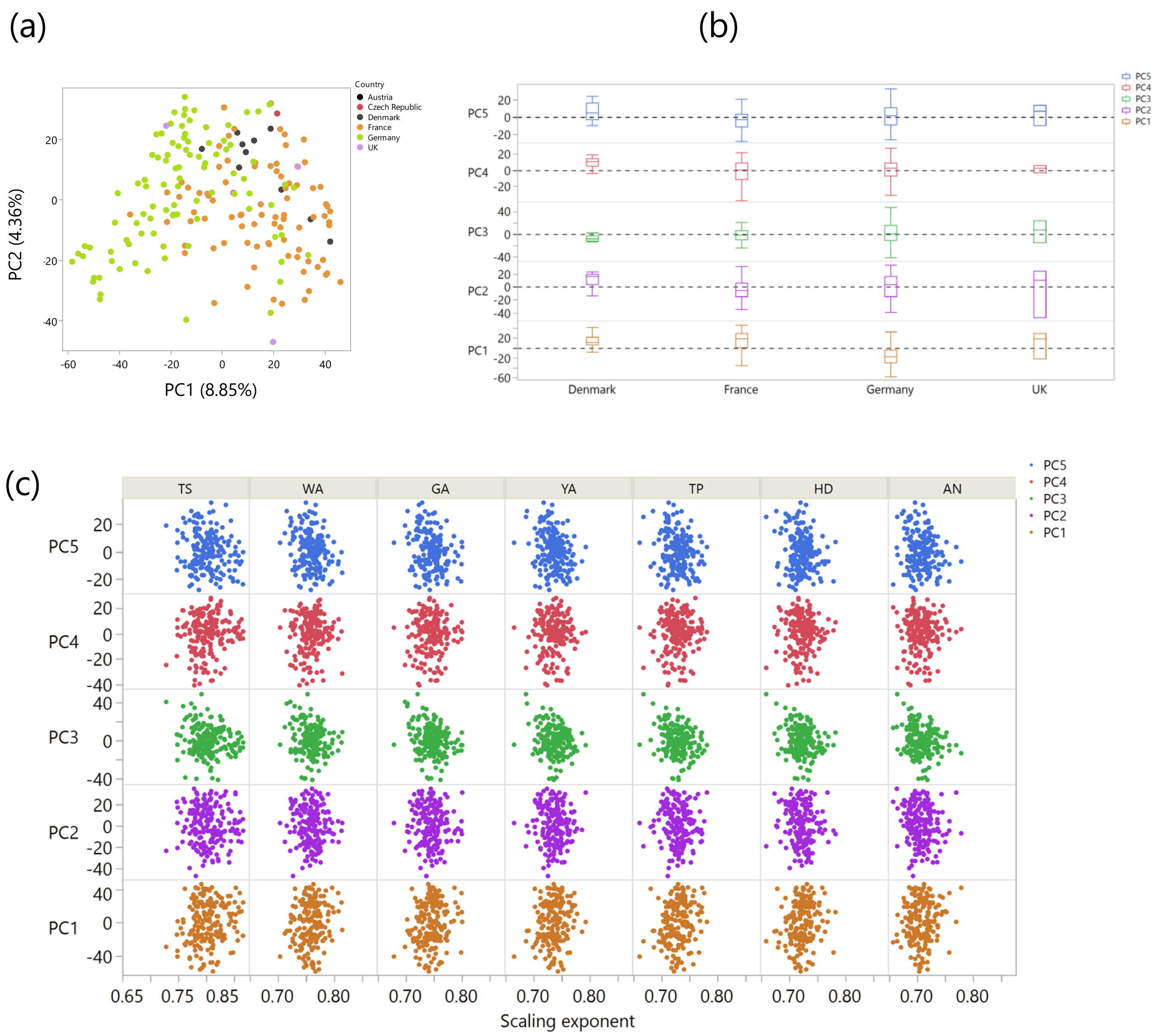


**Fig. S10** Population structure of the GABI wheat panel and its association with the country of origin and the scaling exponent. (a) Biplot of the population structure of the GABI wheat panel. (b) Association between the cultivar’s country of registration and the population structure principal components. (c) Relationships between the scaling exponent at different stages and the population structure principal components.

(a)

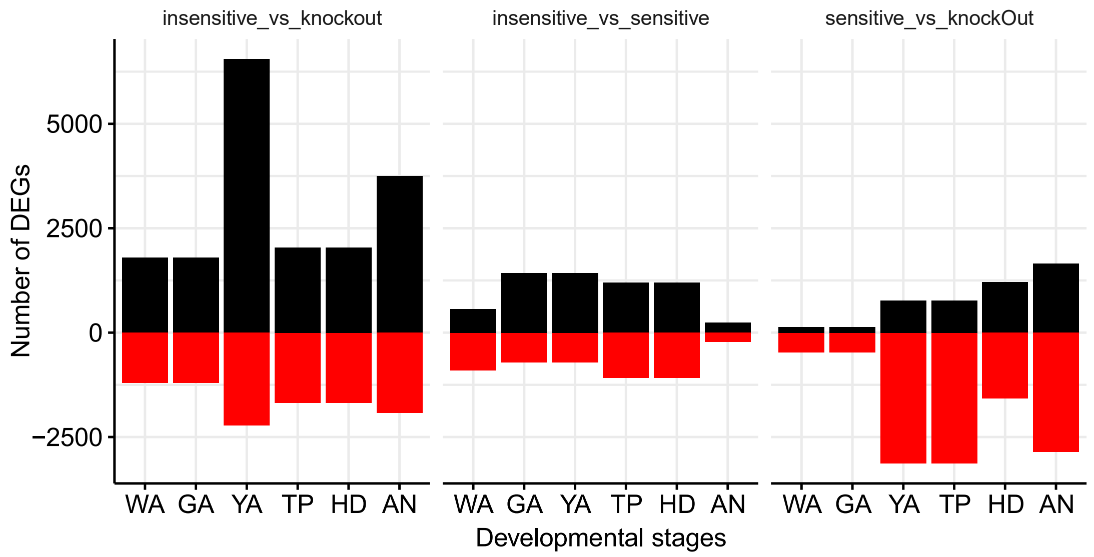


(b)

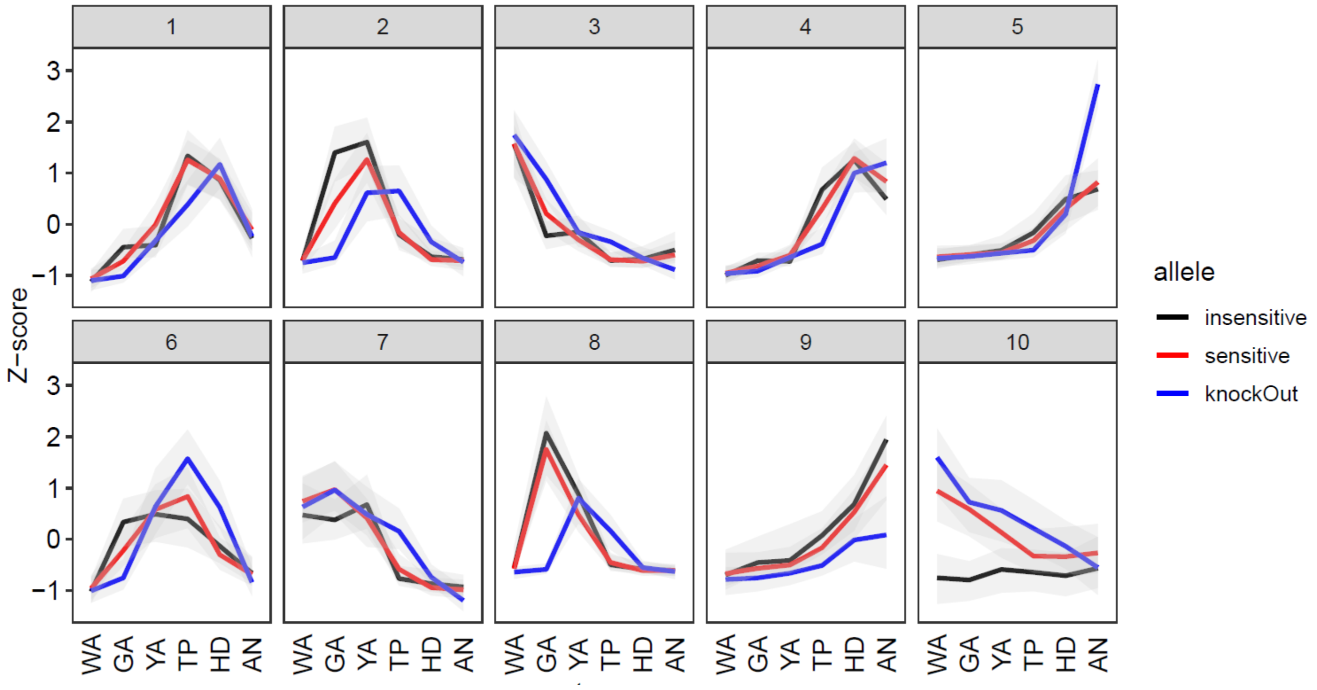


**Fig. S11** Gene expression in the spikelets of near-isogenic lines with differing *Ppd-1* alleles, observed from WA to AN. (a) The number of up regulated and down regulated genes between genotypes at different developmental stages. (b) Clusters of DEGs expression during spikelet growth.
